# Supplementary material for: Epithelial-specific loss of Smad4 alleviates the fibrotic response in an acute colitis mouse model
Source: Life Sci Alliance. 2024 Oct 4;7(12):e202402935. doi: 10.26508/lsa.202402935 (PMC11452480; doi:10.26508/lsa.202402935)
Supplement: Supplementary file 1 [file LSA-2024-02935_TableS1.docx]

Table S1. Primary Antibodies used for immunohistochemistry and immunofluorescence staining.

| <!--Col Count:4-->Antibody | Dilution | Catalog# | Company |
| --- | --- | --- | --- |
| α-Smooth Muscle Actin (D4K9N) XP® Rabbit mAb | 1:500 | 19245 | Cell Signaling Technology |
| BrdU | 1:100,1:1000 | M0744 | Dako |
| COL1A1 (E8F4L) XP® Rabbit mAb | 1:200 | 72026S | Cell Signaling Technology |
| Histone H3 [p Ser10] | 1:1000 | NB21-1091SS | Novus Biologicals |
| E-Cadherin | 1:100 | sc-59778 | Santa Cruz Biotechnology |
| Ki67 | 1:500 | ab16667 | abcam |
| Mouse GPVI Antibody | 1:50 | MAB6758-SP | R&D systems |
| Purified anti-NOS2 | 1:100 | 690902 | Biolegend |
