# Supplementary material for: Epithelial-specific loss of Smad4 alleviates the fibrotic response in an acute colitis mouse model
Source: Life Sci Alliance. 2024 Oct 4;7(12):e202402935. doi: 10.26508/lsa.202402935 (PMC11452480; doi:10.26508/lsa.202402935)
Supplement: Supplementary file 2 [file LSA-2024-02935_TableS2.docx]

Table S2. Secondary antibodies used for immunohistochemistry and immunofluorescence staining.

| <!--Col Count:4-->Antibody | Dilution | Catalog# | Company |
| --- | --- | --- | --- |
| Goat anti- mouse IgG (H+L), Biotinylated | 1:300 | BA-9200 | Vector Laboratories |
| Goat anti- rabbit IgG (H+L), Biotinylated | 1:700 | BA-1000 | Vector Laboratories |
| Alexa Flour 488 goat anti-rat IgG (H+L) | 1:250 | A11006 | Invitrogen |
| Goat anti-mouse IgG Alexa Flour 488 | 1:250 | A28175 | Invitrogen |
