# Supplementary material for: Epithelial-specific loss of Smad4 alleviates the fibrotic response in an acute colitis mouse model
Source: Life Sci Alliance. 2024 Oct 4;7(12):e202402935. doi: 10.26508/lsa.202402935 (PMC11452480; doi:10.26508/lsa.202402935)
Supplement: Supplementary file 3 [file LSA-2024-02935_TableS3.docx]

Table S3. Other reagents and Materials.

| <!--Col Count:3-->Name | Catalog # | Company |
| --- | --- | --- |
| BrdU (5-Bromo-2'-deoxyuridine) | 19-160 | EMD Millipore |
| DAPI | 5087410001 | Sigma-Aldrich |
| DSS 40 kDa | J63606 | Thermofisher Scientific |
| EDTA | 324504-500ML | EMD Millipore |
| EGTA | 0732-10G | VWR |
| Eosin | 95057-848 | VWR |
| Hematoxylin | 26030-20 | Electron Microscopy Sciences |
| HEPES | J848 | VWR |
| Methyl green | ZH0804 | Vector Laboratories |
| NaCl | S271-1 | Fisher Scientific |
| NaF | A13019-30 | Alfa Aesar |
| Sodium Vanadate | 72060 | Sigma-Aldrich |
| Paraformaldehyde | 15714-S | Fisher Scientific |
| PBS | BP399-4 | Fisher Scientific |
| PMSF | P7626 | Sigma-Aldrich |
| Proteases inhibitor | P8340-5ML | Sigma-Aldrich |
| Tamoxifen | T5648-1G | Sigma-Aldrich |
| Triton x-100 | 0694 | VWR |
| TRIzol | 15-596-018) | Thermo Fisher Scientific |
| 70-micron filter | 431751 | Corning |
| ABC-HRP Vectastain kit | PK-4000 | Vector Laboratories |
| BCA Protein Assay Kit | 0023224 | Fisher Scientific |
| CRP assay kit | ab222511 | abcam |
| Click-iTTM Plus Edu Alexa FlourTM 594 Imaging Kit | 2559157 | Thermo Fisher Scientific |
| ImmPACT (TM) DAB HRP Substrate, | SK-4105 | Vector,Laboratories |
| Sirius red/Fast green kit | 9046 | Chondrex |
